# “I’ll Try and Make Myself as Small as Possible”: Women and Gender-Diverse People's Safety Work on Public Transport

**DOI:** 10.1177/10778012241270279

**Published:** 2024-08-06

**Authors:** Jessica Ison, Kirsty Forsdike, Nicola Henry, Leesa Hooker, Angela Taft

**Affiliations:** 1 La Trobe Rural Health School, 2080 La Trobe University, Bendigo, Victoria, Australia; 2 School of Global, Urban and Social Studies, RMIT, Melbourne, Victoria, Australia; 3 Judith Lumley Centre, 2080 La Trobe University, Melbourne, Victoria, Australia

**Keywords:** public transport, sexual violence, sexual assault, women, work

## Abstract

Public transport is a known hotspot for sexual violence and harassment. Through 41 interviews with women and gender-diverse people who have experienced sexual violence and harassment on public transport, we found that women and gender-diverse people engage in extensive “safety work,” such as changing their behavior, strategizing, and planning. Safety work takes considerable time and effort, often leaving participants feeling stressed. However, participants advocated for changes to public transport to reduce the work they do to stay safe. We argue that significant changes, including primary prevention of gender-based violence, are needed to reduce their safety work.

Public transport is a well-known site for sexual violence and harassment (Gekoski et al., 2017; Loukaitou-Sideris & Ceccato, 2020). Women and gender-diverse people are subjected not only to sexual violence and harassment but are also forced to engage in “safety work” (Kelly, 2011) to keep themselves safe or to enhance feelings of safety on public transport. Safety work refers to the labor that women and gender-diverse people undertake to feel safe and remain safe on public transport, such as changing their routes, considering where they sit in a vehicle, or lengthy planning before leaving the house (Newton et al., 2020). Such labor is often invisible and takes a considerable toll on people's sense of well-being and their time (Vera-Gray, 2017, 2018).

To date, there has been little qualitative research into women and gender-diverse people's safety work on public transport. In this article, we sought to explore women's and gender-diverse people's perceptions of safety on public transport and the work they do to feel safe. This article draws on interviews with 41 women and gender-diverse people from two Australian universities. First, the article outlines the literature on sexual violence and harassment on public transport and how women's responses to fear have been theorized. Following this, we make a case for framing the labor as “safety work.” Then, an outline of the context of the study and methods are provided. Our themes are organized across two key areas: “The safety work women and gender-diverse people do,” and “Ideas for reducing the work.” We then offer a discussion and policy implications and limitations. Overall, we argue that safety work on public transport involves a complex interweb of strategies. We recommend that instead of placing the onus on individual public transport users, the responsibility for preventing and reducing sexual violence and harassment on public transport should lie with public transport providers.

## Sexual Violence and Harassment on Public Transport

Sexual violence refers to “any sexual act, attempt to obtain a sexual act, unwanted sexual comments or advances, or acts to traffic, or otherwise directed, against a person's sexuality using coercion, by any person regardless of their relationship to the victim” (World Health Organization, 2021). Sexual harassment, a form of sexual violence, is described in Australian legislation as follows:

any unwelcome sexual advance, request for sexual favors or conduct of a sexual nature in relation to the person harassed in circumstances where a reasonable person would have anticipated the possibility that the person harassed would be offended, humiliated or intimidated. (*Sex Discrimination Act 1984* (Cth))

While sexual violence is an umbrella term that includes sexual harassment, in this article we use the cojoined term “sexual violence and harassment” to bring attention to sexual harassment, which is an often-overlooked form of sexual violence and is sometimes excluded in definitions of sexual violence.

Globally, the World Health Organization (2021) estimates that 6% of women have experienced nonpartner sexual violence in their lifetime since the age of 15. In Australia, one in five women (22%) have experienced sexual violence (defined as sexual assault or threat of sexual assault) since the age of 15, and one in two women (53%) have experienced sexual harassment in their lifetime (Australian Bureau of Statistics, 2021).

People's experience of sexual violence and harassment can differ depending on an intersection of a range of structural oppressions, including racism, sexism, colonialism, trans- or homophobia, and ableism (Townsend et al., 2022). For instance, emerging research on gender-diverse people shows they have experienced higher rates of sexual violence and harassment than their cis-gender counterparts (Hill et al., 2020).

Sexual violence and harassment are known to occur at various “hotspots,” such as universities, workplaces, and on public transport. It is well established that women experience high rates of sexual violence and harassment on public transport (Gekoski et al., 2017; Loukaitou-Sideris & Ceccato, 2020). Furthermore, studies have also focused on different cohorts to understand their experiences, for example, university students, and found a higher prevalence for women compared to men (Australian Human Rights Commission, 2017; Natarajan et al., 2017; Whitzman et al., 2019). Emerging research also indicates that transgender and gender-diverse people experience sexual violence and harassment on public transport (Ison et al., 2023; Lubitow et al., 2017; Santoro et al., 2020).

Sexual violence and harassment on public transport can happen on the platform, on the transport vehicle, or on the “last kilometer home” which refers to the journey from exiting the vehicle to the destination (Chowdhury & Van Wee, 2020; Natarajan et al., 2017). Women are more likely than men to be public transport dependent, meaning they are more likely to need to use public transport than men (Loukaitou-Sideris, 2016). Along with being transport dependent, women may also “trip chain” meaning they may have a more complex use of public transport (de Madariaga, 2012; Levy, 2019). For example, they may have to do household labor such as shopping or taking care of children or elderly parents. Their destinations may not be on a single transport line (Jirón et al., 2020). While many women are impacted by sexual violence and harassment on public transport, some women may be at a heightened risk due to intersecting inequalities, such as racialized or homophobic abuse (Ison et al., 2023; Santoro et al., 2020).

Alongside incidents of sexual violence and harassment, women and gender-diverse people often feel afraid for their safety on public transport. This may be heightened due to fears of physical assault and femicide, particularly when there are well-publicized murders of women on or around public transport (Whitzman et al., 2019). To date, research has focused on women's perception of fear and safety. The research highlights that women have a much greater fear of crime than men (Loukaitou-Sideris, 2014) and that this fear of crime is different from men who may fear theft, rather than sexual violence and harassment (Loukaitou-Sideris & Ceccato, 2020). In the context of public transportation, women's fear has been found to be greatest during the evenings (Ceccato et al., 2022), high-density periods (Beller et al., 1980), or when on an empty platform (Vanier & D’Arbois, 2017). Thus, fear is felt during all parts of the public transport journey (Plan International & Monash University, 2018).

## Precautionary Behavior

Due to feeling afraid, people on public transport may take certain actions to keep themselves safe or to enhance their feelings of safety. The research has tended to focus on women's safety, though feminist and queer geography has looked at how LGBTQ+ people embody space and enact safety (Bell & Valentine, 1995). Public transport research has framed women's actions in response to feeling afraid as “precautionary measures,” which refers to certain strategies that are adopted, such as changing their route, the time they travel, or the areas and routes they may avoid (Loukaitou-Sideris & Samuels, 2009, p. 3). Women enact precautionary measures at higher rates than men (Yavuz & Welch, 2010). For example, Newton et al. (2020, p. 280) assessed what they call “precautionary behavior” on public transport enacted by students. The data come from a larger study of students across 18 countries (Ceccato & Loukaitou-Sideris, 2020). They looked at data from five cities (Guangzhou, London, Los Angeles, Paris, and Vancouver) and when disaggregated (no data on other genders), they noted that “the percentage of females who used precautionary behavior was higher in all cities across all modes of transport” (Newton et al., 2020, p. 289).

While not everyone who takes public transport uses precautionary behavior, those who have had a previously negative experience on public transport are more likely to engage in such behavior (Stark & Meschik, 2018). Regardless of previous experiences, the types of precautionary behaviors that people take are similar across the research (Ceccato & Loukaitou-Sideris, 2022; Jirón et al., 2020; Kash, 2019; Lea et al., 2017; Stark & Meschik, 2018). For example, Newton et al. (2020) report that people may avoid certain stops, always travel with someone else, not wear jewelry that could be used to grab or choke them, and so on. They note that the most common types of strategies are avoidance strategies, including avoiding routes or trying not to take public transport.

## Safety Work

Public transport literature has used the terminology “precautionary behavior” to discuss the actions women take to feel safe on public transport. The research tends to focus on how individual women act rather than thinking about this behavior within the broader context of gender-based violence that women and gender-diverse people are forced to navigate. Outside public transport literature, such actions have been labeled as “labor” or “work.” For example, women's engagement in labor outside of their paid employment has been framed historically as “invisible work” (Fishman, 1978). This can include additional work, particularly emotional labor, which they are expected to provide in their workplace or engagement in certain feminized professions (Hochschild, 2012). Additional work is also carried out by women in the home, particularly for those women in heterosexual relationships, which can include both physical and emotional work (Ciciolla & Luthar, 2019). To date, little has been written about gender-diverse people's additional work.

Discussion of this labor has been extended to consider the work that women are expected to enact to stay safe, often called “safety work.” First coined by Kelly (2011), it refers to the work that has “become an automatic reﬂex, especially when in public space alone as a woman: so automatic that we no longer notice the strategies that we use in our attempts to limit or avoid intrusions” (Vera-Gray & Kelly, 2020, p. 269). This work is often invisible and requires extensive time and effort. Vera-Gray discusses safety work in relation to public spaces (Vera-Gray, 2017, 2018), including public transport, and reflecting on Kelly, states that:

Safety work is hidden because it is related to what constitutes being a woman – not seen as something women do but as something that they are. And this causes problems not only because it renders what we do invisible even to ourselves, but also because it can mean we blame those who don’t act in the ways we feel they should. (2018, p. 82)

The work women undertake can be complex and include both direct and indirect (Bovill et al., 2022) safety work. At the same time, the culture normalizes men's behavior and does not expect safety work from them.

## Safety Initiatives

The focus on individual work also extends to initiatives produced by public transport providers for passenger safety. Feminists for more than 40 years have attempted to raise awareness about sexual violence and harassment on public transport (Guiliano, 1979; Matthewson & Kalms, 2021; Scholten & Joelsson, 2019). While there have been some initiatives to address the issue, progress has been slow and tended toward limited sustained initiatives (Forsdike et al., 2024; Gekoski et al., 2015). There has been increased focus on public transport initiatives going beyond single response-based initiatives to a more primary prevention focus (Ison & Matthewson, 2023). Primary prevention refers to addressing the root causes of gender-based violence (Hooker et al., 2020). This study aims to contribute to the literature on how public transport could be a site of primary prevention.

## This Study

### Study Context

In Australia, public transport is publicly and privately owned and operated. Each state and territory are responsible for its own transport, though there are some rail and bus services that cross state and territory borders. In the state of Victoria, the service Public Transport Victoria is run by the Department of Transport and Planning. Melbourne, the capital city of Victoria and the second largest city in Australia, comprises multiple modes of transport. The train service is run by Metro Trains, the tram service by Yarra Trams, and multiple small to large private bus companies service the city. In regional areas of the state, there are also bus services alongside a regional train line called VLine. The transport can be overcrowded, particularly in peak times in metropolitan areas. However, there are also considerable times when vehicles and platforms may be very sparsely populated, which is even more so in regional areas and late at night.

In terms of safety initiatives for transport users, these have been piecemeal and have limited clear evidence. As with most cities, these include safety access points such as emergency buttons or intercoms, closed circuit television (CCTV), and ticket inspectors called Authorised Officers who only have the power to check tickets. What is distinct about Melbourne is the implementation of Protective Services Officers, colloquially referred to as “PSOs.” Instituted by the Victorian Government in 2011, originally to respond to and prevent crime, PSOs are deployed at metropolitan train stations, trains, trams, and major bus interchanges and select regional stations. PSOs are a branch of the publicly funded Victoria Police, but they are not police officers, though they have similar powers. Originally, they were limited to search and arrest, which made them little more than security guards. Over time, their powers have increased, including now carrying firearms. They are generally only operational on and around train stations, though at times they are deployed to assist with crowd control at major events. Their powers are mostly around arresting, detaining, and searching people, seizing illegal items (i.e., weapons, graffiti tools), issuing infringements, and issuing “move on” directions.

### The Current Study

In 2019, a Palestinian-Israeli woman named Aiia Maasarwe was brutally raped and murdered as she exited a tram to walk back to her on-campus accommodation in Melbourne. Our research was commissioned by the State Government to respond to this atrocity. One element of the research was to conduct in-depth qualitative interviews with women and gender-diverse victim survivors of sexual violence and harassment on public transport who were staff or students at one of two Melbourne universities. The overarching research question for the interviews was: “What are women and gender-diverse university workers’ and students’ experiences of sexual violence and harassment on public transport?”

## Method

### Design

We used semistructured interviews with women and gender-diverse staff and students at two universities in Melbourne, Australia. The Melbourne metropolitan area has multiple universities across the central business district and within the city's inner and outer suburbs, accessible via multiple transport services. The study was approved by the La Trobe University Human Ethics Committee (HEC19293).

### Participants

Participants were recruited through posters, promotion through university email lists, and snowballing. The recruitment poster asked for expressions of interest in participation by women and gender-diverse people who had an “unwanted experience” on public transport. Potential participants emailed the lead author who explained the study and assessed their eligibility and safety to participate, considering the topic's sensitivity. Participants were only eligible if they were female, nonbinary, or gender-diverse. While there was a requirement that participants had to be students and/or staff members at one of the universities, the sexual violence and harassment event or events did not need to have happened on or during their commute to the university, it could have occurred on any type of public transport journey.

In total, we interviewed 41 participants who had a diversity of backgrounds (see Table 1). To indicate ethnicity, participants were given an open text box, with the largest cohort identifying as Australian (*n* = 7). Thirty-two participants identified 19 different ethnicities and two did not specify an ethnicity. The age range was 18–50 years, with the majority (68%) of participants under 30 years.

**Table 1.** Participant Demographic Characteristics (*n* = 41).

*Staff or student*	
Staff member	6
Student	31
Staff and student	4
Residential student	3
International student	7
Aboriginal and/or Torres Strait Islander	1
*Gender*	
Female	39
Nonbinary	1
No gender	1
*Sexuality*	
Heterosexual	28
Lesbian, bisexual, pansexual, and/or queer	12
Prefer not to say	1
Living with a disability	10

### Procedure

Once the first author confirmed participant eligibility, an interview was organized in person or via video conferencing. Before the interview, participants were sent a Participant Information Statement which outlined the risks and benefits of the study and included a list of support services. At the start of the interview, a consent form was signed, including for audio recording. Interviews were semistructured and lasted for up to 1 hour. Participants were asked about their experience or experiences of sexual violence and harassment on public transport, the ongoing impacts, the work they do to keep themselves safe, and their ideas for safety. All research team members had attended training on responding to disclosures of sexual violence and a distress protocol was followed in the interview. At the conclusion of the interview, participants received a $20AUD grocery voucher.

### Data Coding and Analysis

The audio recordings were professionally transcribed. Transcriptions were imported into NVivo 12 (QSR International, 2013), a software program used for qualitative analysis. We followed a reflexive thematic analysis process (Braun & Clarke, 2022). We chose this method to explore the stories of the participants rather than find the truth or an answer. It also offered the opportunity to reflect on our own position and biases as we engaged with the data. We reflected on our position as feminist researchers who specialize in gender-based violence. We also reflected on our identities as cisgender and white, with tertiary education, to consider our biases and assumptions.

To begin, the first author read and re-read the transcripts to deeply engage with the data. The whole team then discussed potential codes, and patterns which we used to construct themes. The themes were not fixed and were in constant reflection as we analyzed the data. We developed four overarching themes. To ensure we covered enough depth, we separated these four themes into two papers. The first article (Ison et al., 2023) reported on the findings of the first two themes: “Experiencing sexual violence on public transport” (with the subthemes “Impact across their lives,” and “Perceptions of reporting”); and “Making sense of the impact” (with the subthemes “emotional impact” and “behavioral impact”). In this earlier article, we noted that sexual violence and harassment are often not one-off incidents, but instead are frequent experiences. While many participants came to the interview to speak predominantly about a single incident, over the course of the interview, participants often discussed other experiences that they viewed as “less serious,” such as being stared at. Participants who experienced other forms of discrimination, such as racism, homophobia, transphobia, or ableism, would often experience sexual violence and harassment in ways that were heightened. The impacts of sexual violence and harassment were felt by participants throughout their lives, not just when taking public transport.

This article focuses on themes three and four: “The safety work women and gender-diverse people do,” and “Ideas for reducing the work.” Quotations below are presented with a pseudonym and any identifying details have been removed or generalized.

## Findings

### The Safety Work Women and Gender-Diverse People Do

Participants employed a range of safety work while using public transport. These are skills they have honed over their lifetime of experiencing sexual violence and harassment. For example, one participant noted experiencing sexual harassment since she was 16 and that those experiences felt more invasive when she was younger “because I’ve created skills on how to cope with using transport, especially at night” (Maya). Some participants said that they were not sure if the safety work was effective, but they noted that they provided a psychological sense of safety.

There were many “safety work” strategies, which can be sorted into three categories: work to change your behavior; transport behavior safety strategies; and safety planning. These strategies often overlapped and informed each other for the participants we spoke to. What stands out with these descriptions is how women and gender-diverse people embody space differently. That is, one person's work was quite different from another person's work. Such diversity was present across all the strategies, which could be contradictory yet there were also some distinct similarities. Participants generally did not realize how much they engaged in safety work until they were asked to elaborate on this during the interviews.

#### Work to Change Your Behavior

The work to change your behavior described by participants fits into four categories (Table 2). First, “gendered norms,” where the participants held an awareness of how they were gendered on public transport, such as their clothing, as well how they should enact gendered norms, such as acting naïve. Second, “body positioning,” where participants thought at length about where they should position themselves and how they should hold their body in the space. Third, “strategizing,” where participants employed a range of strategies to feel safe. And last, “state of mind,” where participants considered not just their physical but also their mental state to remain alert but also how to distract themselves. Table 2 outlines the specific actions participants discussed throughout the interviews.

**Table 2.** Work to Change Your Behavior.

Gendered norms	Positioning body	Strategizing	State of mind
Act dumb or naïve	Move from sitting to standing when afraid	Look for allies/bystanders	Awareness
Do not wear loose clothing that can be pulled	When walking, stay on the path, walk with people, stay in the dark, or stay in well-lit areas	Talk on phone	If feeling anxious, distract your mind with reading
Ensure demeanor is closed off to not attract people but not make them angry	Stand/sit near security area, panic button, driver, or other safety area	Wear headphones but play no music	Hypervigilance
Make sure hair is not in ponytail that can be pulled	Sit near other women or gender-diverse people	Improvise weaponry (i.e., keys in knuckles of hand)	Remain present
Respond nicely if someone talks to you but try to end the conversation	Ensure the body is prepared to fight or escape (i.e., do not cross legs)	Tell others your location or send them location on an app	Rumination
	Never make eye contact	Have an escape plan	Self-consciousness
	Sit near the door	Do not talk to anyone	

Gendered norm behavioral change included complying with binary gender norms such as acting nice as well as being compliant. However, it also included ensuring that they did not act in any way that they thought men would see as an invitation to interact with them, such as having a closed-off demeanor. While some participants found that the best strategy was to be present, such as Areeba saying, “I mean just make sure that I’m looking around and smiling at people,” others found it better to try and make themselves invisible:

I’ll try and make myself as small as possible, so I’ll put my bag underneath my feet. I’ll keep myself, not quite hunched over, but as lower profile as I can be and slip my headphones on and try not to move. Just kind of like fade into the rest of the people just going home. (Giorgia)

Participants also considered how to dress in ways that would minimize any possibility for grabbing their hair or clothing: “I’ll put my hair in a low bun as opposed to a ponytail or out where it can be grabbed” (Lara). Thus, participants considered how to stay safe within socially proscribed gendered norms but also how to use these gendered norms to feel safe.

In terms of body positioning, participants mentioned the ways that they sit, stand, and how they hold their bodies. For example, when boarding a vehicle, participants often look for other women, as well as checking on the safety of other women. Often, participants chose to sit next to a woman where possible: “Yeah. I think I always am very aware of where I am and who I’m sitting in relation to. I’ll always try and stick with other women” (Sarah).

Strategizing included the considerable work undertaken to plan a public transport journey and the strategizing that occurred while on the journey. Participants discussed looking for bystanders, but their strategies predominantly focused on individual actions such as having an escape plan or improvising weapons:

I used to carry a heavy steel water bottle, now I just have a plastic one, but I used to do that, you know, the keys between the knuckles thing, I don’t think that would be effective really so I don’t really do that, it's more just a placebo sort of thing if you feel safe. (Lara)

While participants were strategizing, they were also focused on their state of mind. They discussed remaining vigilant and present: “I guess it's kind of like an unconscious thing that is like happening in my head a lot of the time if I’m going to or from places especially in the dark” (Isla).

Yet conversely, some participants chose to “zone out” by reading a book or looking at their phone to allow some distance for themselves over the journey. For example, Chloe said she will often read because “I think that's, you know, a protection mechanism that a lot of people would use. Just having your attention focussed on something else.”

Not all participants employed all strategies or actions, though they generally employed some or many. Rachel captures this well in her description:

I don’t wear makeup on public transport. I tie my hair back in a way that it adds 10 years to me. I generally wear pants. I walk from and to stations with keys in my hand. And like I’ve had pretty substantial training in forms of self-defense, like, I know how to take care of myself, but I still do all of these things. I watch where I sit. I don’t cross my legs because it's easier to get up. I make sure that I don’t drink before I get on a train or that I don’t drink on the train because if I drink on the train, then as any woman who's hit 30 will tell you, you’ll need to pee. And if you need to pee, then you end up having to use like one of the unmanned station's toilets…

The number of strategies utilized by Rachel shows that even bodily functions need to be controlled to ensure safety.

#### Transport Behavior Safety Strategies

The second safety category was transport behavior strategies, which included: “planning,” where participants planned ahead before taking public transport, and “avoiding,” where participants chose to avoid public transport. Table 3 outlines the specific transport behavior safety strategies participants discussed throughout the interviews.

**Table 3.** Transport Behavior Safety Strategies.

Planning	Avoiding
Alter route each trip	Take a taxi or rideshare
Travel with others	Have someone pick you up
	Don’t walk
	Ride bike instead
	Drive everywhere
	Do not attend event

Participants enacted a range of planning work practices when they were on their journey. Planning could include altering their route every time to ensure that no one was able to monitor their activities and stalk them. As Isla explained: “If I’m on an unfamiliar route, like I very, very much plan my journey. And like I leave plenty of time and I like make sure have my journey up on maps and on the PTV [Public Transport Victoria] app and have like back up plans and everything. I can’t just wing it.”

Participants also employed avoidance safety work strategies. As Poppy stated: “I just try to actively avoid any kind of situation where I might be unsafe.” The way that they navigated the transport system often resulted in such compromises of not attending events or having to pay for a rideshare: “Look, I don’t go out by myself at night” (Savannah)

#### Safety Planning

The third safety planning category was safety planning that happened before a journey. This included: “collective” engagement, where participants discussed the knowledge they shared collectively, and “individual” planning, where participants discussed their individual planning work. Table 4 outlines the specific safety planning participants discussed throughout the interviews.

**Table 4.** Safety Planning.

Collective	Individual
Organize place to stay after a night out	Plan a trip with minimal transfers
Share knowledge with friends to be prepared	Make sure you’re not running late so you don’t rush and as a result don’t get a safe seat
	Always plan your route
	Have a backup route
	Live near public transport so don’t have to walk far

The collective action of participants mostly involved how they share the safety work and information on how to stay safe:

There's always conversation about keeping… safe. [We ask each other] What time? How will you? Who will you be with? In terms of getting home and getting on transport, there's always that undercurrent and that checking, “Okay, well where's the tram stop? How long is the next tram? When will you be? Who's on it? Where will you sit?” (Sarah)

As with much of this safety work, they focused on nighttime as a particularly important point to make alternative plans.

Individual planning work shows how the fear of public transport interrupts other elements of their lives. Women and gender-diverse people could decide to not take public transport somewhere, or they had to consider all elements and plan each aspect, including a backup route: “I think the big ones are just planning ahead and more or less just taking control of my own whereabouts to the biggest extent that I can” (Tara).

### Ideas for Reducing the Work

Participants tended to individualize the work they enacted to stay safe. When asked about how to reduce the safety work they do, they tended to see the issue as needing to go beyond individual peoples’ labor. This mostly involved changes to the transport system, though some participants also spoke about the need for broader cultural change.

#### Public Safety Officers (PSOs)

Some participants said they would like to see more PSOs on public transport, including trams and buses as well. The remaining participants either did not want more PSOs or did not mention them. There is considerable misinformation about PSOs, for example, some participants did not know that PSOs are predominantly on train platforms. Those who liked the PSOs might advocate for more PSOs, such as Fatima who said: “I like that the Victoria government provide police officers in each train station. This is good. But what about around the train station? Or walking to the train station.” While some participants did suggest more PSOs, others were skeptical and referred to concerns about the PSOs: “PSOs I just see as standing on the platform, they don’t actually really make me feel any safer because I don’t feel like if someone's going to target me it's going to be on the platform” (Lara).

Other than PSOs, participants mentioned the need for increased security, including in the form of conductors on public transport. Previously in Victoria, trams had conductors and participants recalled them with fondness and advocated for their return, although they were clear that having security on every transport vehicle was unrealistic.

#### Communication

The need for clearer communication and dedicated communication campaigns came up throughout the interviews. Interviewees explicitly discussed how much they would appreciate a dedicated communication campaign about sexual harassment on public transport. Beatrice explained this as “signs or posters around [the transport network], maybe because if people see it and read it on their journey, then it's still in the back of their mind somewhere.” Similarly, Lara talked about posters, saying:

I feel like maybe something like posters at the station, things that are more direct, like something that's catchy but pretty much says “Don’t talk to women on public transport, they don’t want you to”, you know, like maybe “Don’t hit on anyone on public transport” and “Don’t be a bigot”, but like in a catchy way for people who want to pick up.

While participants suggested campaigns around women's safety, the messaging also needed to include consumer diversity. For example, Djamila advocated this in terms of religious and cultural diversity: “I would like to see a flyer that has a woman wearing hijab and whatever the topic about, I would feel that's normal in this, in this, in this country, on this city and other people.”

#### Bystanders

In our first article (Ison et al., 2023), we reported on how participants discussed not having any assistance from other transport users and how this was part of their negative experience. It was not that they were harmed but rather that no one came to their aid. Therefore, participants advocated for better bystander behavior. For example, Jamie said:

I think we could all be better bystanders … You know, because being a good bystander doesn’t mean going up against someone who's threatening, it could be an ally to the person who's being threatened or, you know, there are, sort of, different things that we could do in society to better support without necessarily inflaming the situation.

The participant focus on bystander activity could also be impacted by a communication campaign (including television advertisements and social media) that was running at the time of the interviews. It was a campaign on sexual harassment from the primary prevention organization RESPECT Victoria (2019). In this campaign, a man on the train is staring at a woman. She is visibly uncomfortable. Another man acts as a bystander by blocking the man's view of the woman. The message was “RESPECT women: Call it out.” Participants talked about this campaign positively. For example, Maya said:

I think it's good. I actually think it's really good. I’m really proud of that because actually it's a man and a man. Can you please sort your shit out between your own gender? I think that a lot.

Similarly, Sara said “I liked that because I thought yep, there's, there's men taking responsibility for men like, in a way. It's like, we are taking responsibility for each other.” However, there have been some online critiques of the RESPECT bystander campaign for focusing on making a man the “hero” and giving women limited agency (Reynolds, 2019).

#### Effective Trip Chaining

Participants also discussed the issue of “trip chaining,” though they may not have known this terminology. Due to participants taking multiple modes of transport, they would often feel fear at transfer points. One solution to this was a system that worked better for trip chaining. For example, Diwa talked about how often two buses will come at one time and:

So if you miss both of them, then you’re shit out of luck for the next 15 minutes. It's not like an alternating thing, where if there was one [redacted suburb] bus, and then a little bit later there's another one, and a little bit later there's another one, and then a little bit later there's another one. No, they both go to the station at the same time, and then you have to catch both.

Similarly, Isla pointed out that she might sometimes wait 40 min if she just missed multiple buses that came at once. Extended waiting periods, particularly when unexpected, left them feeling vulnerable, particularly during the evening. A significant delay on one part of the journey could delay their whole journey and leave them at multiple stations or stops where they usually plan to spend as little time as possible.

Participants proposed the solution of staggered buses that worked more effectively for trip chaining. Alongside the effectiveness of trip chaining, was the frustration over transport systems stopping quite early in some parts of Melbourne. In particular, bus routes may finish before 9 pm, making it difficult for participants to get home without paying for a rideshare or walking a long distance from another mode of transport. If they were a participant with a disability, walking may not be an option and therefore rideshares became very costly. They talked about the need for later bus services.

#### Women's Only Transport

One idea discussed by participants was women-only carriages. Participants mentioned those that exist in Japan and India. However, there was some ambivalence to this idea:

I think that would be interesting way of doing things. I would feel safer totally more relaxed, I think it would be very good. But I don’t think that's the answer, I think that's just, you know, making the segregation worse. (Olivia)

The feeling that women-only carriages would cause segregation was echoed by others. It was also raised that such a gendered approach could exclude people: “it's not really helpful in terms of like for me not all people who say, you know ‘look like women’ and experience misogynistic harassment are actually women and things like that” (Lara).

#### Cultural Change

Participants also discussed the need for cultural change, though they acknowledged from the outset that this was a large issue:

So it probably comes down to the really kind of tough and maybe a bit sucky answer of a broader culture change … I can understand that's not a great answer because that's something that takes time and it's really hard to do. (Matilda)

Part of this is a need to focus less on quick fixes and more on men's behavior change. Participants noted that when the issue gains government and media attention—often in light of a particularly heinous event such as the murder of Aiia Maasarwe—there are a lot of promises to make changes, but these are mostly not long term. Sarah voiced frustration at the focus on quick fixes:

So that made me just feel frustrated that here we are just throwing more surface, you know, stuff and not really addressing men, men's violence and you know, men's rage and their behavior towards women and you know, all genders really that aren’t theirs. You know that there was nothing about what we’re going to be rolling out new programs. But you know, there was nothing about that. There was nothing to kind of try and bring men around to the conversation. It was all about lights and cameras. So that felt really like, you know, we’ve been missing these opportunities for so many decades. So, none of that stuff makes me feel any better.

To deal with this, Sarah went on to say: “But actually, men being responsible for their own behavior and how their behavior can be perceived. Yes. It wouldn’t necessarily immediately make me feel safer, but I would feel like we were all on the pathway together, actually.”

Part of this cultural change was the need to focus on prevention. As Triana said:

If they built some more lights and stuff around the tram stops, which is great, but that's not going to…and CCTV but then what? Then it happens and then what can they do? They can’t do anything to help you. It's just proof. They might capture the offender or whatever and then yeah, but then it's already happened and someone else is traumatized or even killed. So, that's more for after.

## Discussion

Interviews with 41 women and gender-diverse people indicate that considerable change is needed to improve the public transport experience for women and gender-diverse people. Beyond “precautionary behavior,” safety work is the work women and gender-diverse people undertake to feel safe when taking public transport. Reconsidering this as a form of active work situates this as work within the broader context of gender inequality. Beyond just a set of behaviors, this is work that takes considerable time, effort, and mental load (Newton et al., 2020; Yavuz & Welch, 2010). To reconsider this as a form of work brings attention to the significant impact this has on women and gender-diverse people as well as the loss of time and income that accompanies such work. It also opens the possibility for expanding the intersectional analysis of safety work, to consider beyond the work women do to include gender-diverse people who have similar and unique forms of safety work.

Our interviews reveal considerable safety work that is needed to plan the public transport journey, such as multiple buses and long wait times, as well as the work involved while taking public transport. This also included the significant burden of deciding whether to avoid going out or deciding to rely on a car, if this was financially available. Avoidant behavior indicates significant insecurity in the transport system for women and gender-diverse people.

As discussed in our initial publication (Ison et al., 2023), participants experienced sexual harassment from a young age, citing being as young as 10 and in school uniform. Such harassment and assault occurred throughout their life and countless times on public transport. Therefore, while research has indicated that those who have experienced sexual violence and harassment were more likely to enact safety work (Stark & Meschik, 2018), it is also clear that such learned behavior occurs from a young age. This is beyond being a reactive behavior to something socially taught and expected. Participants often started off with one or two examples of the work they do to stay safe but with gentle prompting, participants would generally start voicing further work they do, which is normalized and everyday, that they rarely think of it as additional work. It is simply something they *have to do*. Vera-Gray (2018, p. 5) discusses this realization she had when interviewing her participants, stating “Despite the commonality, or perhaps because of it, we rarely even think about the things we do. We don’t talk about the habitual, sometimes unconscious, choices and changes we make daily to maintain a sense of safety in public space.” Such habitual and unconscious safety work was clearly part of our participants’ general life and travel behavior and was often exacerbated if they experienced other forms of violence, such as racism or homophobia.

Participants had suggestions about how public transport could be made safer. They tended to look beyond just the actions of individuals to situate their ideas within the broader cultural context. However, some suggestions need to be considered with caution. For example, there is currently little research on women-only carriages (Dunckel-Graglia, 2013), and those countries that employ this strategy differ from Australia. Referred to as “pink” transportation, they are occasionally suggested as a solution in countries such as Australia and are often met with backlash by feminists. For example, in the United Kingdom, Jeremy Corbin in 2015 suggested this as a solution, and in Australia, the Rail, Tram, and Bus Union secretary suggested pink transport in 2016. Such suggestions face scrutiny, particularly in the media where journalists and public commentators have called it “ridiculously regressive” (Jackson, 2016) or “bonkers” (Bindel, 2022). The public discourse against such ideas sees it as reinforcing the idea that the onus of women's safety is on the victim rather than the perpetrator. There is also concern that women who are not in a women-only carriage will then be blamed for their victimization. Also, there is concern about the binary nature of such suggestions, which could exclude trans and gender-diverse travelers (Moss, 2022). In the interviews, participants cited some of these concerns and reflected on the limitations of this suggestion.

Some participants spoke about wanting more PSOs, though most either did not reference the PSOs or, as reported in our earlier findings (Ison et al., 2023), were critical of the PSOs. This is reflected in the broader research on PSOs. They have faced considerable controversy, including multiple reports highlighting their ineffectiveness at reducing crime (Victorian Auditor-General, 2016) and their troubling overreach including sexually harassing young women (Victorian Ombudsman, 2015). PSOs were deployed to make public transport safer and to reduce crime, yet the overwhelming negative or indifferent response to them indicates that participants did not see them as effective.

Participants also discussed the role of bystanders. However, it was clear in their responses that bystanders could not be relied on, even though at the time of the interviews there was a popular communication campaign relating to bystanders. Evidence indicates that people often do not intervene but that if and/or when they do intervene, such as coming to talk with the victim, it is often positive for the victim (Whitzman et al., 2020). The participants’ experiences contribute to the bystander literature to indicate that bystander approaches should be explored with caution.

While participants were cognizant of their own safety work and had suggestions for what could be enacted by public transport, they were at times despondent about public transport. They tended to have a view that things were not likely to change, and this was particularly heightened when they experienced multiple intersecting forms of oppression. However, there is the potential for public transport to be a key site of change, particularly primary prevention (Ison & Matthewson, 2023). Such a change would need sustained attention and would need to shift the onus away from the focus on safety work by individual women and gender-diverse people.

## Policy Implications

Our research highlights people's experience of sexual violence and harassment across the life course, how this impacts women and gender-diverse people, and the complex gendered work they employ to remain safe. Participants’ suggestions for how to improve safety show the need to listen to women and gender-diverse peoples’ varied needs and consider complex changes beyond easy fixes.

Our research also focused on misogynistic advertising present at public transport locations, the need for improved public transport data collection, increased gender-based violence training for public transport staff and management, and design-specific improvements (TramLAB, 2020). Alongside early intervention such as bystander campaigns, we also advocate for a shift to primary prevention. Instead of focusing on individual risk strategies, primary prevention looks at preventing perpetration (Hooker et al., 2020). We recommend that public transport becomes a key site for implementing primary prevention programs (Ison & Matthewson, 2023), and that an intersectional lens is required to address sexual violence and harassment.

## Limitations

This study was limited to one city in Australia and did not include rural areas. It also focused on university staff and students. The interviews were conducted in English. Future research should focus on diverse cohorts outside of the university context. Our sample was diverse in terms of ethnicity and a mix of staff and students, with many identifying as lesbian, bisexual, pansexual, and/or queer. However, the majority were cisgender women. Future research should consider how to expand to recruit a larger number of trans and gender-diverse participants.

We also note that focusing on safety work could reinforce women and gender-diverse people as “weak” or always “at risk.” Indeed, the extensive safety work discussed by participants would indicate that such work is always at the forefront of their minds. We offer two important considerations. First, the participants came to an interview to discuss a personal experience or experiences of sexual violence and harassment on public transport and were thus victims of sexual violence and harassment who were actively thinking about their experiences. Therefore, this may not reflect the experiences of all women and gender-diverse people at *all* times. Second, discussing the work undertaken by women and gender-diverse people does indeed show the troubling aspects of their daily lives navigating public transport but it can also indicate the active steps people take to feel some agency against what can be perceived or experienced as an ever-present threat. The complexity of navigating safety work shows how agency and power are neither static nor simple.

## Conclusion

Sexual violence and harassment on public transport is both a lived reality as well as something women and gender-diverse people fear. As a result, they enact safety work to both be safe and feel safe. While participants had strategies that helped them to feel empowered as well as effective strategies that had worked for them over their lifetime, they felt some anger at having to do this safety work. Urgent action is needed to address sexual violence and harassment on public transport. A shift to primary prevention would mean that there was a focus on preventing perpetration, thereby reducing the safety work women and gender-diverse people are forced to undertake to keep themselves safe on public transport.
